# Oct1/Pou2f1 is selectively required for colon regeneration and regulates colon malignancy

**DOI:** 10.1371/journal.pgen.1007687

**Published:** 2019-05-06

**Authors:** Karina Vázquez-Arreguín, Claire Bensard, John C. Schell, Eric Swanson, Xinjian Chen, Jared Rutter, Dean Tantin

**Affiliations:** 1 Department of Pathology and Huntsman Cancer Institute, University of Utah School of Medicine, Salt Lake City, UT, United States of America; 2 Department of Biochemistry, University of Utah School of Medicine, Salt Lake City, UT, United States of America; 3 Howard Hughes Medical Institute, Salt Lake City, Utah, United States of America; Stockholm University, SWEDEN

## Abstract

The transcription factor Oct1/Pou2f1 promotes poised gene expression states, mitotic stability, glycolytic metabolism and other characteristics of stem cell potency. To determine the effect of Oct1 loss on stem cell maintenance and malignancy, we deleted Oct1 in two different mouse gut stem cell compartments. Oct1 deletion preserved homeostasis in vivo and the ability to establish organoids in vitro, but blocked the ability to recover from treatment with dextran sodium sulfate, and the ability to maintain organoids after passage. In a chemical model of colon cancer, loss of Oct1 in the colon severely restricted tumorigenicity. In contrast, loss of one or both *Oct1* alleles progressively increased tumor burden in a colon cancer model driven by loss-of-heterozygosity of the tumor suppressor gene *Apc*. The different outcomes are consistent with prior findings that Oct1 promotes mitotic stability, and consistent with differentially expressed genes between the two models. Oct1 ChIPseq using HCT116 colon carcinoma cells identifies target genes associated with mitotic stability, metabolism, stress response and malignancy. This set of gene targets overlaps significantly with genes differentially expressed in the two tumor models. These results reveal that Oct1 is selectively required for recovery after colon damage, and that Oct1 has potent effects in colon malignancy, with outcome (pro-oncogenic or tumor suppressive) dictated by tumor etiology.

## Author summary

Colorectal cancer is the second leading cause of cancer death in the United States. Approximately 35% of diagnosed patients eventually succumb to disease. The high incidence and mortality due to colon cancer demand a better understanding of factors controlling the physiology and pathophysiology of the gastrointestinal tract. Previously, we and others showed that the widely expressed transcription factor Oct1 is expressed at higher protein levels in stem cells, including intestinal stem cells. Here we use deletion of a conditional mouse *Oct1* (*Pou2f1*) allele in two different intestinal stem cell compartments to study gut homeostasis. We then proceed to investigate the effect of Oct1 loss in colon regeneration and malignancy. The results indicate that Oct1 loss is dispensable for maintenance of the mouse gut, but required for recovery after damage to the colon epithelium. We also find that Oct1 loss has opposing effects in two different mouse colon cancer models, and further that the two models are associated with different gene expression signatures. The differentially expressed genes are enriched for Oct1 targets, suggesting that differential gene control by Oct1 is one mechanism underlying the different outcomes.

## Introduction

Oct1/Pou2f1 is a widely expressed POU domain transcription factor related to the embryonic stem cell master transcription factor, Oct4 [1, 2]. Oct1 promotes glycolytic metabolism and mitotic stability [3–5]. It also promotes poised gene expression states, i.e. the ability of transcriptionally silent target genes to be readily induced in response to different cues [6, 7]. Oct1 loss is associated with increased oxidative metabolism, elevated reactive oxygen species, hypersensitivity to oxidative and genotoxic stress, and a modest increase in abnormal mitoses [3, 4, 8–10]. Oct1 loss does not compromise cell viability, affect immortalization by serial passage or reduce growth rates in standard culture, however oncogenic transformation in soft agar is strongly reduced [3]. In a well-characterized *Tp53* null mouse model, loss of even one Oct1 allele suppresses thymic lymphoma [3]. In a variety of malignancies, Oct1 motifs are enriched in coordinately activated genes [11–14]. These results indicate that Oct1 plays important roles in stress responses and tumorigenicity.

We and others have shown that Oct1 promotes somatic and cancer stem cell potency in different systems [9, 15, 16]. For example, in the blood system Oct1 loss allows for primary hematopoietic engraftment but compromises serial transplant capacity [9]. Despite the connection between Oct1, stem cells and oncogenic potential, *Oct1* (*Pou2f1*) mRNA is not elevated in stem cells [17]. Instead, Oct1 is regulated post-translationally, e.g. at the level of DNA binding specificity [5], sub-nuclear localization [4, 10, 18–20], and protein stability [15, 21]. Regarding the latter, two Oct1 ubiquitin ligases have been identified, Trim21 and BRCA1 [15, 21]. A third activity, CKIP-1, negatively regulates Oct1 by enhancing its association with the proteasome activator REGγ [22]. Although Oct1 is widely expressed, Oct1 protein levels are elevated in mouse and human gastric, small intestine and colon stem cells [9, 23, 24]. In malignancy, multiple studies identify a correlation between tumor aggressiveness and elevated Oct1 expression [9, 11, 21, 25–31], including in the GI tract [24, 32–34].

Here, we use a conditional Oct1 allele together with two gut stem cell Cre-drivers to determine the role of this protein in the maintenance and regeneration of normal gut cells, and in colon malignancy. The Cre-drivers are tamoxifen-inducible to provide both tissue-specific and temporal control over Oct1 deletion. We find that Oct1 is dispensable for colon homeostasis, but required for the colon epithelium to recover from injury. In the small intestine, Oct1 deletion from Lgr5^+^ cells in vivo allows for the establishment of cultured gut organoids from isolated crypts, but not their maintenance following passage. In a chemical model of colon tumorigenesis, Oct1 loss greatly diminishes tumor incidence and size. The tumors that do emerge in this model have escaped Oct1 deletion, indicating a requirement for Oct1. In contrast, a model of colon cancer driven by loss-of-heterozygosity (LOH) shows a progressive increase in tumor number, though not of grade, as one or both *Oct1* alleles are lost. Comparison of gene expression signatures in the two models identifies a set of genes correlating with the model of origin, indicating that the two models have distinct molecular features. This set is enriched in direct Oct1 targets identified using ChIPseq in human colon cancer cell lines.

## Results

### Oct1 is dispensable for colon epithelium homeostasis

**Fig 1:**
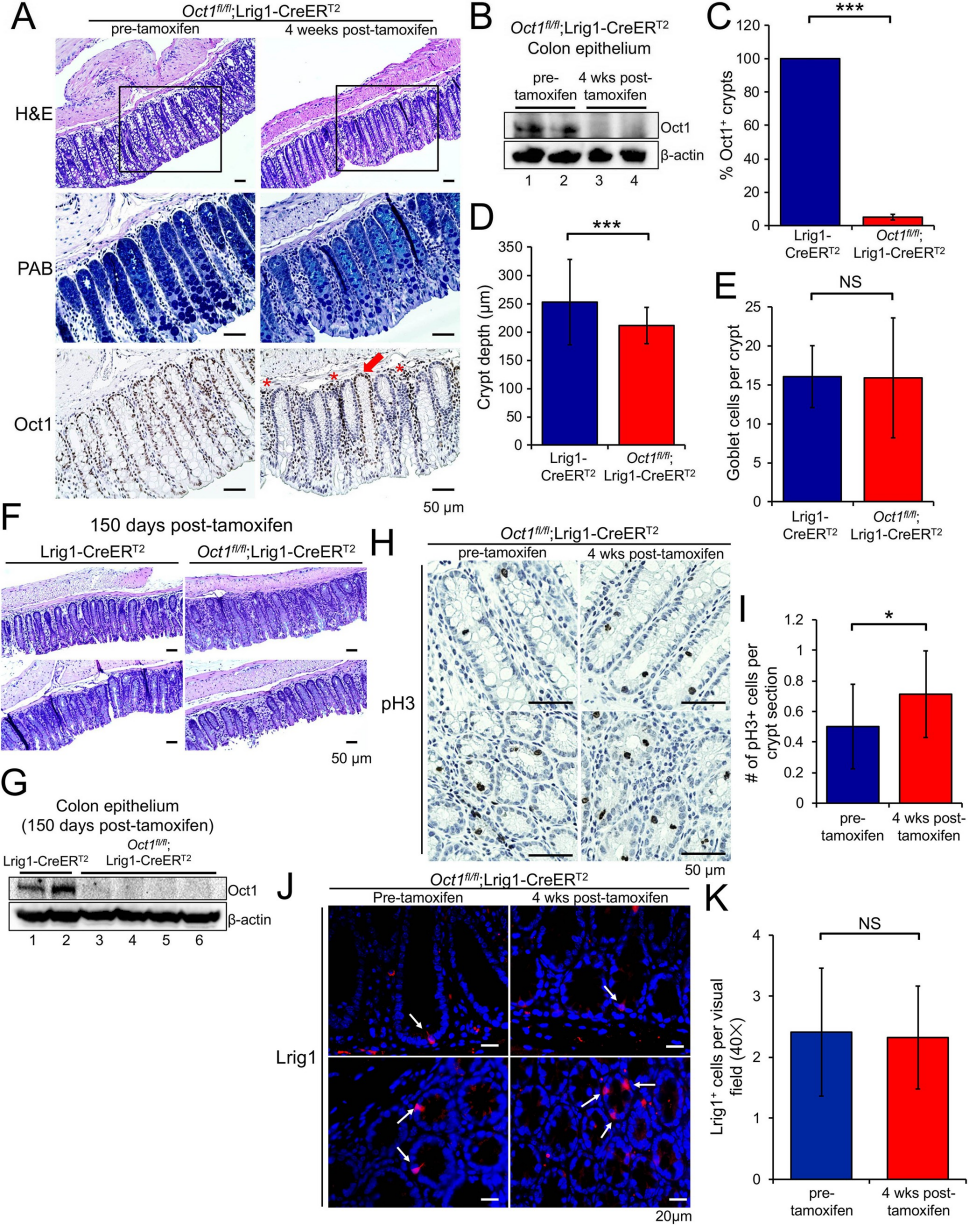
Loss of Oct1 maintains normal colon homeostasis. (A) Lrig1-CreER^T2^;*Oct1*^*fl/fl*^ mice were treated with tamoxifen to induce the deletion of Oct1 in the Lrig1^+^ cells of the colon. Representative H&E and Alcian blue stained images of the distal colon, as well as IHC using Oct1 antibodies, are shown. Adjacent sections of the same tissue were used. The box within the H&E panels indicates the area of adjacent section magnified in the adjacent Alcian blue and Oct1 IHC images. (B) Colon epithelial cells from mice sacrificed prior to or 4 weeks post-tamoxifen treatment were isolated and subjected to immunoblotting using anti-Oct1 antibodies. β-actin was used as a loading control. (C) Averages of Oct1 IHC-positive crypts from five Lrig1-CreER^T2^ control and five Lrig1-CreER^T2^;*Oct1*^*fl/fl*^ experimental mice are shown. (D) Quantification of crypt depth. 79 and 78 crypts were analyzed from three Lrig1-CreER^T2^ control and three Lrig1-CreER^T2^;*Oct1*^*fl/fl*^ experimental mice, respectively. Only crypts that were visible longitudinally were used. (E) Similar analysis as in (D), quantifying goblet cells based on intense Alcian blue staining. (F) Representative H&E stained images of the distal colon 150 days post-tamoxifen treatment. (G) Colon epithelial cells from mice sacrificed 150 days post-tamoxifen treatment were isolated and subjected to immunoblotting using anti-Oct1 antibodies. β-actin was used as a loading control. (H) Colon sections from Lrig1-CreER^T2^;*Oct1*^*fl/fl*^ mice were subjected to IHC using pH3 antibodies. Representative images from mice pre- and 4 weeks post-tamoxifen treatment are shown. (I) Quantification of pH3 staining. 20 and 21 images from Lrig1-CreER^T2^ control and Lrig1-CreER^T2^;*Oct1*^*fl/fl*^ experimental mice, respectively, were analyzed. (J) Colon sections from Lrig1-CreER^T2^;*Oct1*^*fl/fl*^ mice sacrificed prior to or 4 weeks post-tamoxifen treatment were subjected to IF using Lrig1 antibodies. Representative images taken at 40× magnification are shown. (K) Quantification of Lrig1 staining. 30 and 31 images from 3 control pre-tamoxifen and 3 experimental post-tamoxifen mice, respectively, were analyzed.

Oct1 is widely expressed in colon epithelial cells, but is more strongly expressed in cells at the crypt base that express Lrig1, which marks the stem cell compartment [9, 35]. We crossed an *Oct1* (*Pou2f1*) conditional allele [6] to Lrig1-CreER^T2^ [35], which upon tamoxifen treatment (single injection of 2 mg in corn oil by gavage, see methods) induces Cre activity in stem cells of the colon. Four weeks after treatment, the colon epithelium was superficially normal despite ~90% deletion of Oct1 (Fig 1A). Oct1 immunoblot using isolated colonic crypts and quantification of Oct1 immunohistochemistry (IHC) from multiple mice confirmed global deletion (Fig 1B and 1C). Non-epithelial cells in the same tissue sections retained staining (Fig 1A, asterisks). There were crypt structures that escaped deletion (red arrow), but these were rare indicating that Oct1 deletion efficiency was higher than reported previously for *Rosa26*-LacZ reporter mice [35]. Crypts lacking Oct1 appeared normal compared to adjacent ones that had escaped deletion and compared to those from mice prior to tamoxifen treatment (Fig 1A, bottom). Quantification of crypt depth from multiple mice indicated a small but statistically significant decrease (Fig 1D). In contrast, quantification of goblet cell numbers based on Alcian blue staining showed no difference (Fig 1E). Oct1 deletion and normal colonic architecture could be maintained for 150 days (Fig 1F and 1G).

To study proliferation in this tissue, we performed phospho-histone H3 (pH3) IHC on tissue from mice pre-tamoxifen and four weeks post-tamoxifen. There was little difference in proliferation in crypts lacking Oct1 (Fig 1H and 1I). To study the stem cell compartment, we performed immunofluorescence (IF) using Lrig1 antibodies. We found that under homeostatic conditions, Lrig1 intensity and numbers of Lrig1^+^ cells were normal (Fig 1J and 1K), consistent with the normal architecture of the colon over long periods. Overall, these results show that Oct1 loss has minimal effect on the colon epithelium under homeostatic conditions.

### Oct1 is dispensable for small intestinal homeostasis

**Fig 2:**
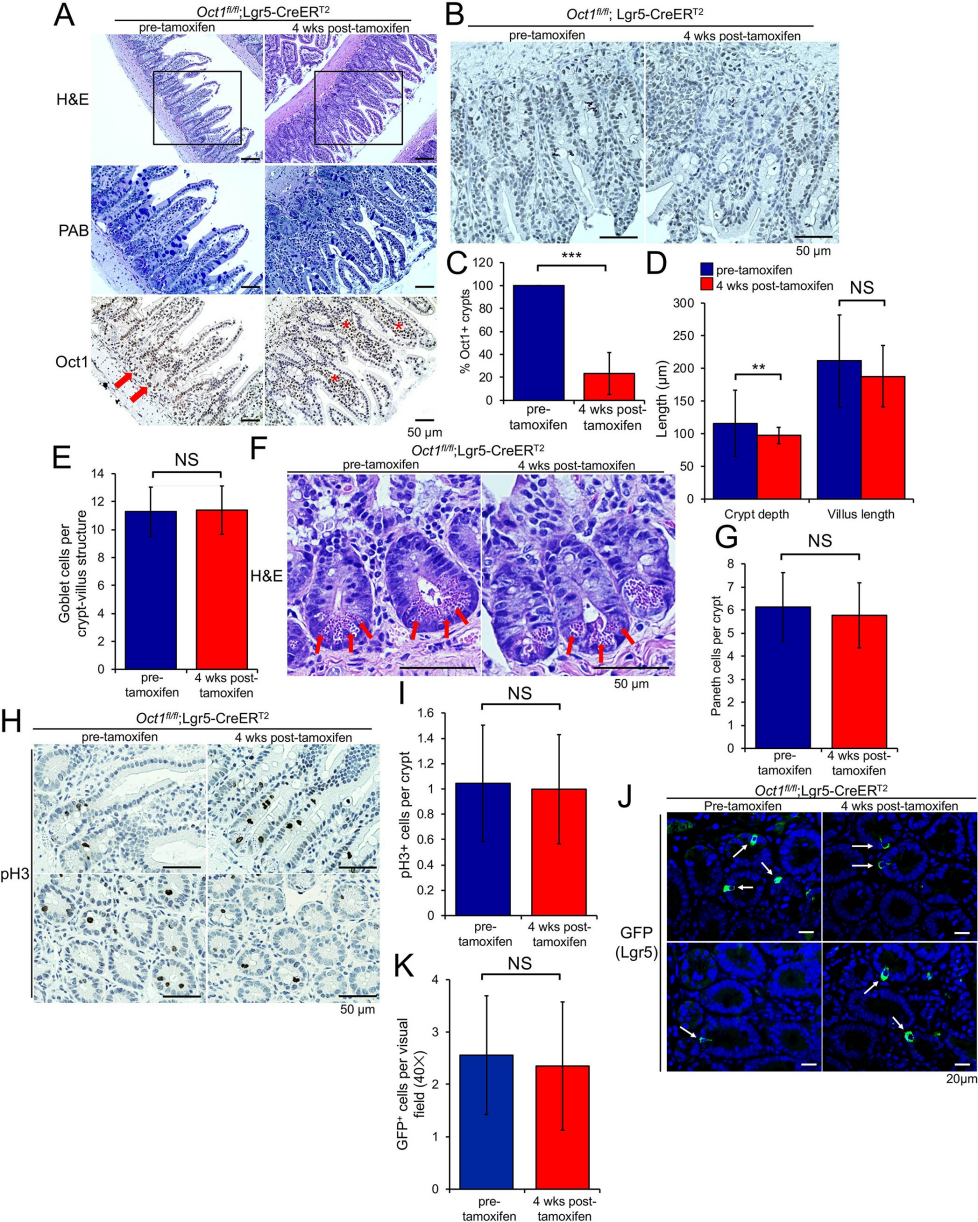
Oct1 is dispensable for the maintenance of the small intestine under homeostatic conditions, but required to maintain organoids *in vitro*. (A) Lgr5-EGFP-IRES-CreER^T2^;*Oct1*^*fl/fl*^ mice (n = 6) and Lgr5-EGFP-IRES-CreER^T2^ controls (n = 4) were treated with tamoxifen for 4 weeks. Representative H&E and Alcian blue images of the duodenum, as well as IHC using Oct1 antibodies, are shown. Adjacent sections of the same tissue were used. The box within the H&E panels indicates the area of adjacent section magnified in the Alcian blue and Oct1 IHC images. (B) Higher-magnification Oct1 IHC images focused on duodenal crypts, where staining was strongest. (C) Quantification of average Oct1 IHC deletion in duodenal crypts from four pre-tamoxifen control (234 crypts total) and six post-tamoxifen experimental *Oct1*^*fl/fl*^;Lgr5-CreER^T2^ mice (236 crypts total). (D) Quantification of crypt and villus length from four pre-tamoxifen control (118 crypt-villus units) and six post-tamoxifen experimental *Oct1*^*fl/fl*^;Lgr5-CreER^T2^ mice (125 crypt-villus units). Duodenum was used. (E) Similar analysis of goblet cell numbers using Alcian blue staining (118 and 125 crypt-villus units from pre and post-tamoxifen mice, respectively). (F) Representative high-magnification H&E images of crypt bases showing Paneth cells using *Oct1*^*fl/fl*^;Lgr5-CreER^T2^ mice pre- and post-tamoxifen treatment. (G) Quantification of Paneth cell numbers from four pre-tamoxifen (112 crypts) and six post-tamoxifen-treated *Oct1*^*fl/fl*^;Lgr5-CreER^T2^ mice (117 crypts). (H) pH3 IHC images from example pre- and post-tamoxifen-treated mice. Arrows indicate example mitoses. (I) Quantification of pH3 numbers per crypt in 1183 crypts from two pre-tamoxifen-treated control *Oct1*^*fl/fl*^;Lgr5-CreER^T2^ mice and 1380 crypts from three tamoxifen-treated experimental *Oct1*^*fl/fl*^;Lgr5-CreER^T2^ mice. (J) Small intestine sections from *Oct1*^*fl/fl*^;Lgr5-CreER^T2^ mice sacrificed prior to or 4 weeks post-tamoxifen treatment were subjected to IF using GFP antibodies. Representative images taken at 40× magnification are shown. (K) Quantification of GFP staining. 34 and 31 images from 3 control pre-tamoxifen and 3 experimental post-tamoxifen mice, respectively, were analyzed.

To determine if Oct1 behaves similarly in the small intestine, we deleted Oct1 using Lgr5-EGFP-IRES-CreER^T2^ mice [36]. These mice express tamoxifen-inducible Cre and GFP under the control of the native *Lgr5* gene, which is expressed in stem cells of the small intestine. *Lgr5* encodes a R-Spondin receptor that stabilizes Frizzled receptors, allowing for increased Wnt signaling [37]. As with the colon, deletion of Oct1 in the small intestine preserved a normal-appearing small intestine in H&E and Alcian blue, despite efficient deletion from the epithelium (Fig 2A). Although IHC is not very quantitative, prior to tamoxifen treatment we observed weaker Oct1 staining in the small intestine compared to the colon. Staining was more robust in cells at the crypt base (arrows) and tapered off in the villi (Fig 2A, arrows, Fig 2B). Quantification of Oct1 deletion from four pre-tamoxifen and six post-tamoxifen-treated animals confirmed robust Oct1 deletion with tamoxifen (Fig 2C). Consistent with the normal appearance of the small intestine, quantification of average crypt and villus length and goblet cell numbers revealed few differences (Fig 2D and 2E). We also identified Paneth cells at the crypt base in pre- and post-tamoxifen treated mice (Fig 2F, arrows). Across multiple mice, no differences in Paneth cell numbers per crypt were identified (Fig 2G). To study proliferation in the deleted small intestine under homeostatic conditions, we performed pH3 IHC from mice pre- and post-tamoxifen treatment (Fig 2H). Consistent with the normal size of the crypts and villi, no differences in mitoses were noted (Fig 2I). Lgr5 staining intensity and numbers of cells were also similar with and without tamoxifen (Fig 2J and 2K). Cumulatively, these results indicate that Oct1 loss results in minimal differences in small intestinal architecture under homeostatic conditions.

### Oct1 is required to maintain passaged organoids in culture

**Fig 3:**
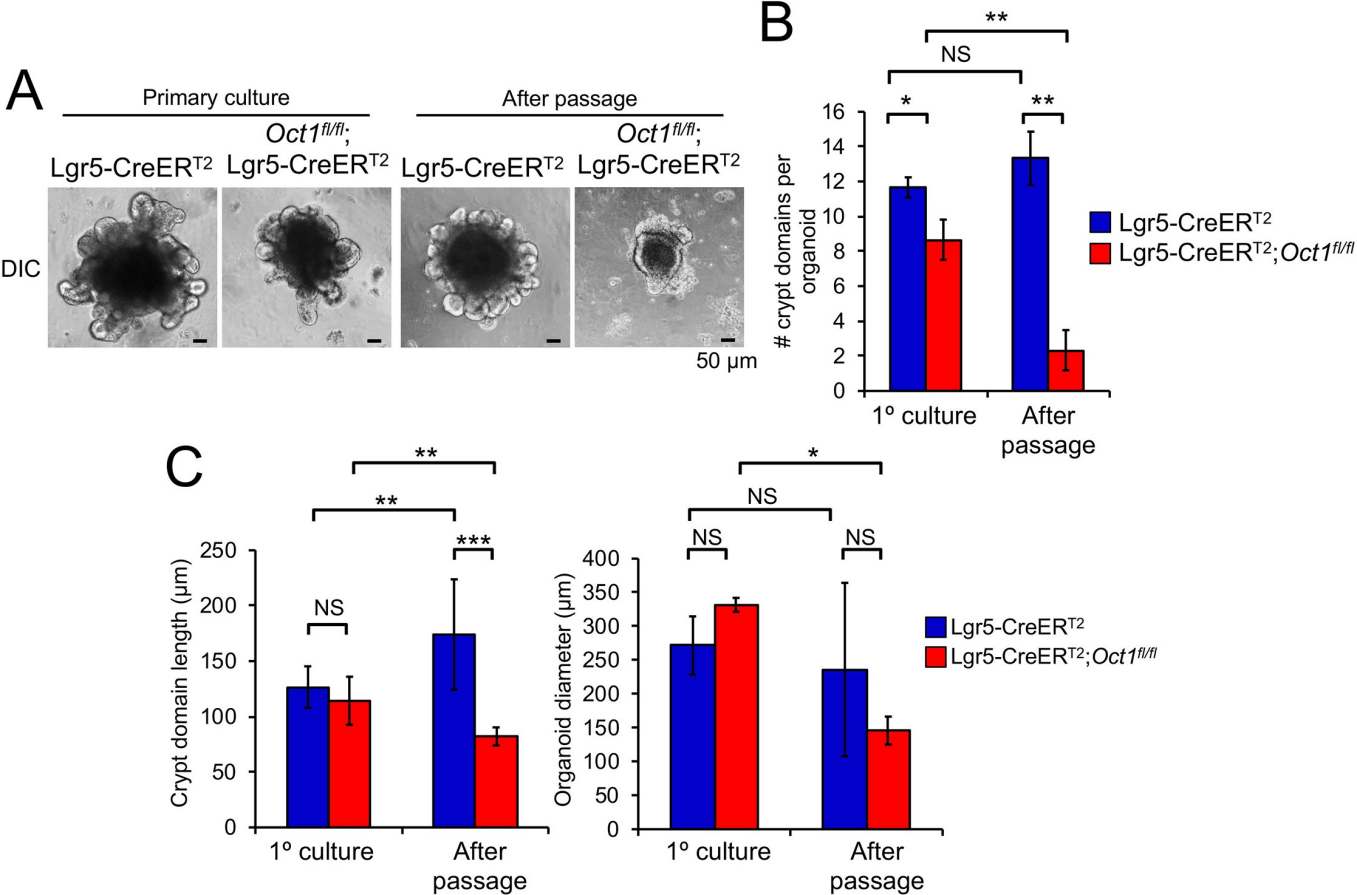
Oct1 is required to maintain gut organoids *in vitro*. (A) Tamoxifen-treated mice were used to form intestinal organoids in culture. Images are shown of primary organoids (left) and after one passage (right). (B) Quantification of the average number of crypt domains per organoid from two mice of each genotype (5 organoids/condition/mouse). (C) Similar analysis for average crypt domain length and organoid diameter.

DSS models are not widely used in the small intestine, as typical treatments generate less damage compared to the colon [38]. To determine whether Oct1 has effects in the small intestine under non-homeostatic conditions, we used cultured small intestinal organoids. In vitro cultured intestinal organoids are a powerful tool to study intestinal stem cells and their differentiated progeny. They are relatively easy to culture and manipulate [36, 39]. We profiled the effect of Oct1 loss in the small intestine using organoids from mice 4 weeks post-tamoxifen treatment. Organoids were generated directly ex vivo from intestinal crypts. No difference in size, viability or morphology were noted (Fig 3A, primary culture). However, upon passage *Oct1*^*fl/fl*^;Lgr5-EGFP-IRES-CreER^T2^, disaggregated crypts were unable to regenerate new villus and crypt structures to generate complete organoids (Fig 3A, after passage). In contrast, control Lgr5-EGFP-IRES-CreER^T2^ organoids grew normally following passage. Quantitatively, these changes manifested most strongly as a reduction in the numbers of crypt domains per organoid after passage (Fig 3B). Reductions in crypt length and organoid diameter were also noted in passaged Oct1 deficient organoids (Fig 3C).

### Oct1 is required for colon epithelium recovery from DSS-induced damage

**Fig 4:**
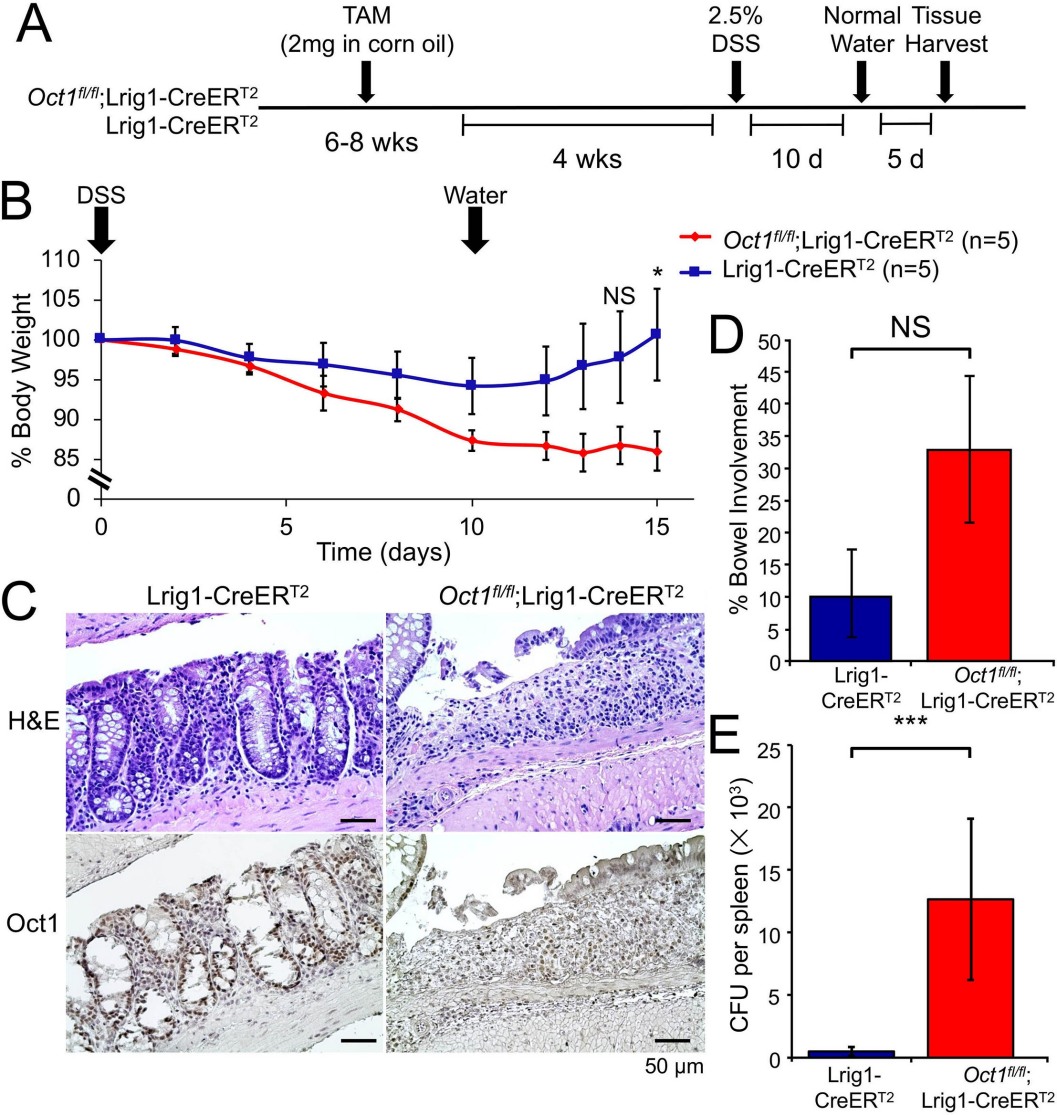
Loss of Oct1 renders the mouse colon more susceptible to DSS-induced damage. (A) Experimental schematic. Lrig1-CreER^T2^;*Oct1*^*fl/fl*^ mice and Lrig1-CreER^T2^ controls were treated with tamoxifen. 4 weeks post-tamoxifen treatment, mice were treated with 2.5% DSS in their drinking water for 10 days. All mice were female littermates. (B) Average body weight changes of the mice (n = 5 for each group from a single representative experiment) were monitored following DSS treatment. Error bars denote ±SEM. (C) Representative Oct1 IHC images of distal colon sections from mice following 10 day treatment with DSS and 5 day treatment with water. Above shows H&E stained adjacent sections of the same tissue. (D) Quantification across the entire colon (excluding cecum) of 5 mice in each group were evaluated for colitis involvement. Average involvement is shown. (E) Spleen homogenates from the same mice as in (D) were grown on blood agar plates to determine bacterial burden and loss of gut barrier function. Average CFUs are shown. N = 5 for each group.

To test if Oct1 play a role in gut regeneration, we next treated *Oct1*^*fl/fl*^;Lrig1-CreER^T2^ mice and Lrig1-CreER^T2^ control mice with 2.5% DSS to damage the GI tract and mobilize gut stem cells to regenerate the epithelium (Fig 4A). Mice lacking Oct1 in their colon trended towards greater sensitivity to DSS treatment, though this was not statistically significant (Fig 4B, day 0–10). Upon switching from DSS to water at 10 days, control mice Oct1 rapidly began to gain weight, while mice lacking Oct1 in the colon continued to lose weight (Fig 4B, day 12–15) and did not recover an epithelial layer (Fig 4C). The failure to regenerate was associated with increased colitis (Fig 4D) and loss of barrier function with microbial infiltrate in the spleen (Fig 4E). Collectively, the data indicate that the colon epithelium does not recover from DSS-mediated damage in the absence of Oct1.

### Oct1 loss protects mice from AOM-DSS-mediated colon tumors

**Fig 5:**
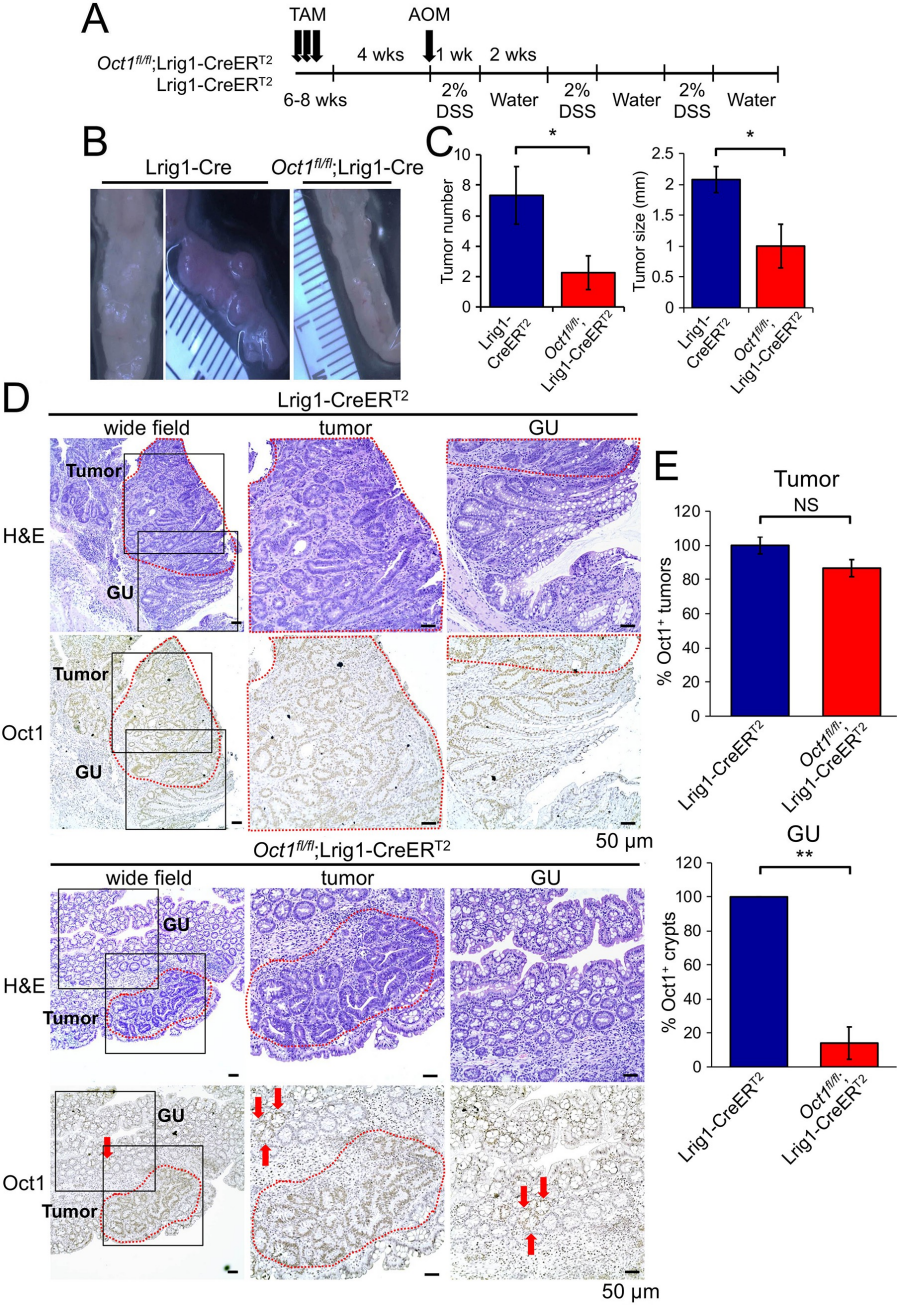
Oct1 loss protects mice from tumor progression in a model of colon cancer induced by AOM/DSS. (A) Lrig1-CreER^T2^;*Oct1*^*fl/fl*^ mice (n = 6) and Lrig1-CreERT2 controls (n = 4) were treated with tamoxifen followed by a single IP injection of AOM (10mg/kg) 4 weeks later. Mice were then exposed to 3 cycles of 2% DSS in their drinking water for 1 week followed by 2 weeks of water. (B) Images of distal colon tumors from representative mice. (C) Quantification of tumor number and size. (D) H&E and Oct1 IHC of colon tumor tissue and gross uninvolved (GU) adjacent tissue. (E) Analysis of Oct1 IHC in tumor and GU tissue.

Colon cancer mimics a chronically regenerating state in many respects [40, 41]. To test if Oct1 loss protects mice from malignancy, we used *Oct1*^*fl/fl*^;Lrig1-CreER^T2^ mice together with a chemical carcinogenesis model driven by the DNA alkylating agent azoxymethane (AOM) [42, 43] (Fig 5A). Colon tumors were efficiently generated in control mice but were fewer in number and smaller in size using Oct1 deleted mice (Fig 5B). Quantification from 6 experimental and 4 control mice is shown in Fig 5C. Control Lrig1-CreER^T2^ tissue showed robust Oct1 expression in both tumor and gross uninvolved (GU) areas (Fig 5D). However, the low-grade *Oct1*^*fl/fl*^;Lrig1-CreER^T2^ tumors that did occur showed a higher proportion of tumor tissue that retained Oct1 expression compared to GU tissue in the same section (Fig 5D). Quantification from multiple mice indicated that ~90% of tumor tissue retained Oct1 staining, while only ~10% of GU tissue did so (Fig 5E). This result indicates a selection against Oct1 deletion, consistent with Oct1 promotion of tumorigenicity in this model.

### Oct1 restricts tumorigenicity in a model of colon cancer driven by loss-of-heterozygosity

**Fig 6:**
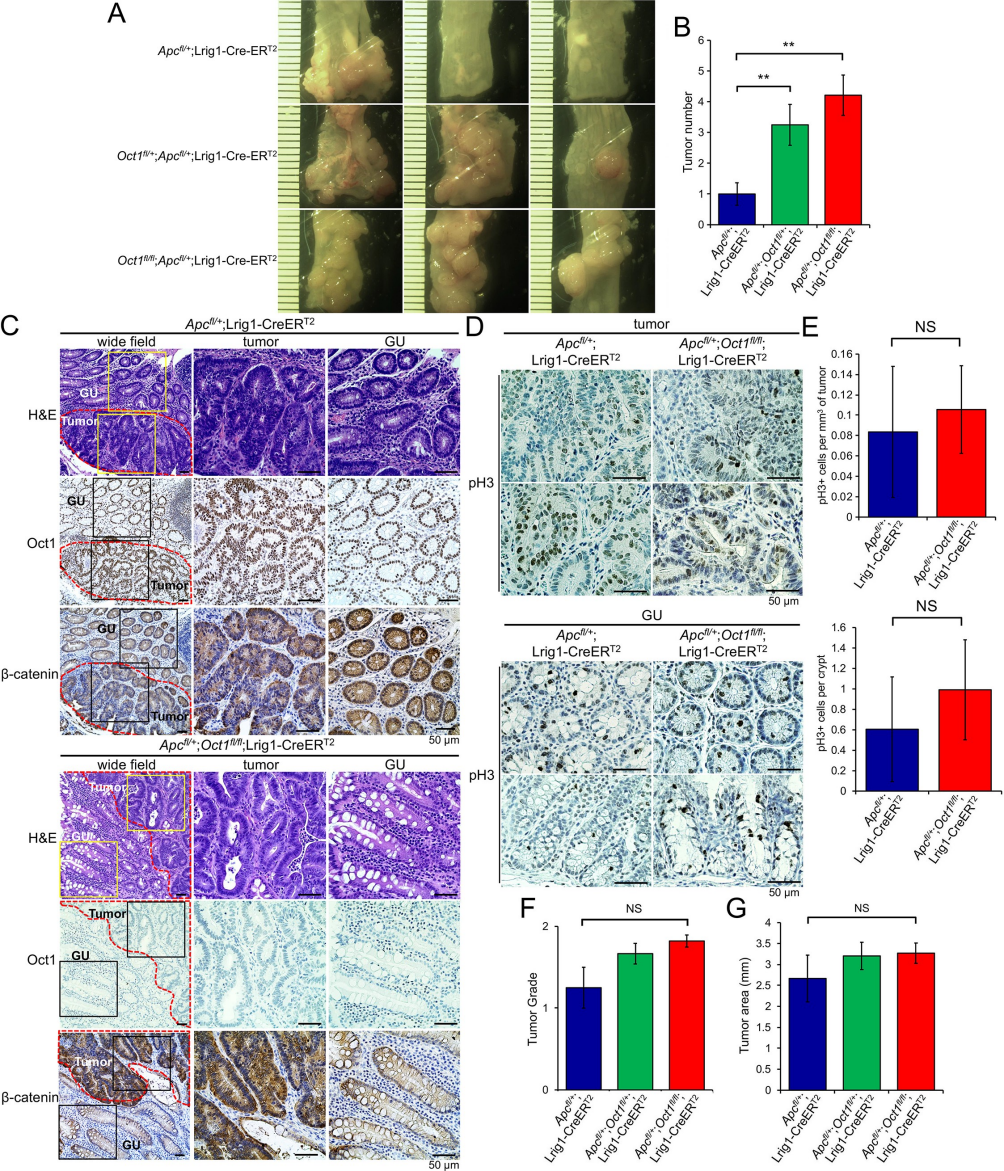
Oct1 restricts tumorigenicity in a model driven by heterozygous *Apc* deletion in Lrig1^+^ cells. (A) Example distal colons are shown of an allelic series of *Oct1*^*+/+*^, *Oct1*^*fl/+*^ and *Oct1*^*fl/fl*^ animals that were additionally *Apc*^*fl/+*^ and Lrig1-CreER^T2^. (B) Quantification of colon tumor incidence across multiple animals. *Oct1*^*+/+*^ N = 14, *Oct1*^*fl/+*^ N = 12 and *Oct1*^*fl/fl*^ N = 6. (C) Representative H&E, and Oct1 and β-catenin IHC of colon tumor tissue and adjacent GU tissue from *Apc*^*fl/+*^;Lrig1-CreER^T2^ and *Apc*^*fl/+*^;*Oct1*^*fl/fl*^;Lrig1-CreER^T2^ animals. Mice were analyzed 100 days after tamoxifen treatment and *Apc*/*Oct1* deletion. (D) Tumor mitoses were studied using pH3 IHC. (E) Quantification of pH3 staining events in tumor and GU tissue from 3 mice per group (10 fields per animal). (F) Quantification of tumor grade across multiple animals. *Oct1*^*+/+*^ N = 14, *Oct1*^*fl/+*^ N = 12 and *Oct1*^*fl/fl*^ N = 6. Tumor grade was low in all cases. (G) Similar analysis performed for tumor area.

Oct1 functions physiologically not to promote tumors, but rather to promote stem cell potency. The stem cell properties that Oct1 promotes are largely pro-oncogenic, but in one respect Oct1 can be tumor suppressive: like its paralog Oct4 [44], Oct1 promotes mitotic stability in some systems [4]. Mitotic stability is a hallmark of stem cells [44–46]. To test the hypothesis that Oct1 loss can accelerate tumor initiation in models of malignancy dependent on mitotic errors and LOH, we used conditional deletion of the *Apc* gene, which is mutated in a large proportion of human colon cancers [47]. Over time, *Apc* LOH results in adenocarcinomas in the distal colon that mimic human disease in many respects [35]. We crossed *Apc*^*fl*^ mice [48] with *Oct1* (*Pou2f1*) conditional mice, generating an allelic series of *Oct1*^*+/+*^, *Oct1*^*+/fl*^, and *Oct1*^*fl/fl*^ Lrig1-CreER^T2^ mice with heterozygous *Apc*^*fl*^. Mice were followed for 100 days post-tamoxifen treatment before sacrifice. Progressive deletion of one or both *Oct1* alleles progressively increased tumor number in this model (Fig 6A). Quantification of H&E sections from five animals confirmed that tumor number was increased (Fig 6B). Using Oct1 IHC we found that Oct1 was again efficiently deleted from normal (gross uninvolved) crypts (Fig 6C), however in contrast to AOM-DSS-mediated tumors, Oct1 was also deleted in most tumor cells (Fig 6C). We also assessed total β-catenin by IHC. Apc protein restricts β-catenin by ubiquitin-mediated degradation [49], and hence *Apc* LOH would be predicted to augment β-catenin specifically in tumors in this model. As expected, we observed accumulated β-catenin, including nuclear β-catenin, in the tumor cells in both control and Oct1-deleted tissue (Fig 6C).

To study tumor aggressiveness in the control and knockout tumor and adjacent GU tissue, we performed pH3 IHC. The number of mitotic events was low, and equivalent in the presence or absence of Oct1 (Fig 6D). Across multiple animals, no significant differences were noted in mitoses per unit area in tumor tissue, or mitoses per crypt in GU (Fig 6E). Consistent with this result, pathological scoring of tumor sections from *Oct1*^*+/+*^, *Oct1*^*+/fl*^, and *Oct1*^*fl/fl*^ Lrig1-CreER^T2^ mice with heterozygous *Apc*^*fl*^ indicated that despite the increased tumor incidence with Oct1 knockout, there were no significant differences in tumor grade or area (Fig 6F and 6G).

### Genes that are differentially expressed between chemical and Apc-LOH tumors are enriched for Oct1 targets

**Fig 7:**
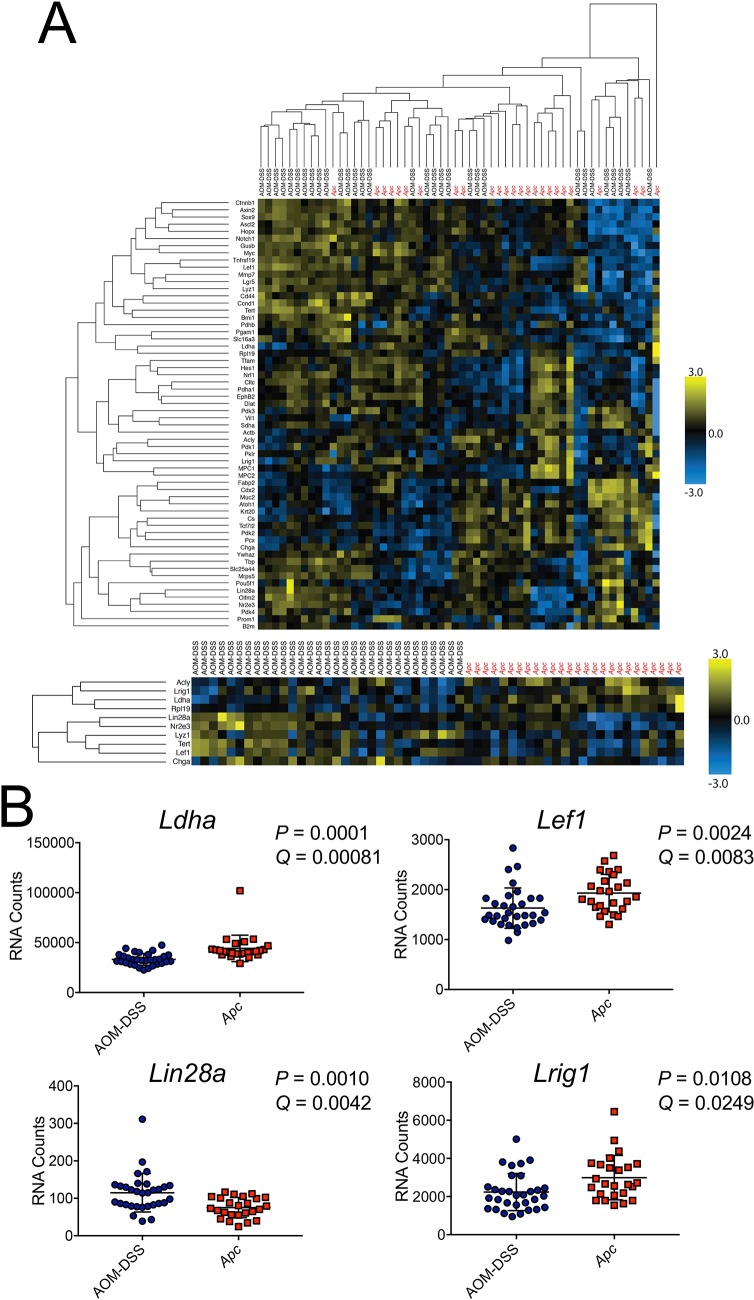
Gene expression profiling reveals a signature of genes that are differentially expressed between the two tumor models. (A) Gene expression analysis performed using a custom probeset with 31 AOM-DSS tumors (23 FFPE, 8 snap-frozen) and 25 *Apc*^*fl/+*^-LOH tumors (12 FFPE, 13 snap-frozen). Top: A heatmap is shown using unsupervised hierarchical clustering using all genes in the probeset. Bottom: Forced clustering by tumor type, showing top clustering of genes based upon mean change and ranked adjusted *P*-value. (B) The overall expression level per tumor sample is shown for example genes (*Ldha*, *Lef1*, *Lin28a*, *Lrig1*) that show significant differential expression based on tumor type.

The finding that Oct1 loss resulted in opposing effects in the chemical- vs. *Apc*/LOH-driven colon cancer models suggested that the two models may differ at a molecular level, such that Oct1’s dominant activity can switch from pro-oncogenic to tumor suppressive. To test this hypothesis, we sampled gene expression from control Oct1 wild-type FFPE and frozen tumor samples. We used a custom 60-gene panel enriched in gut-associated stem cell function [50] together with 31 AOM-DSS-induced tumor samples (23 FFPE and 8 frozen), and 25 *Apc*^*fl*^;Lrig1-CreER^T2^ tumor samples (12 FFPE and 13 frozen), all of which were wild-type for Oct1. The output data is shown in S1 Table. Unsupervised hierarchical clustering of gene expression resulted in interdigitation of the samples (Fig 7A, top). The interdigitation was robust using multiple settings and cutoffs, indicating that tumor-to-tumor gene expression variation is too great to cluster by model using all the genes in the probeset. To identify model-associated molecular signatures, we performed supervised clustering, grouping samples by the model-of-origin and applying an FDR cutoff of 0.05. This analysis identified a subset of the profiled genes whose expression partitions with the model. A group of 17 genes was identified whose expression tended to correlate with tumor model (Fig 7A, bottom). Examples are shown in Fig 7B. The differentially expressed gene list is shown in S1 Table, with and without an additional fold-change cutoff applied reducing the cohort to 10 genes.

To further test the robustness of this result, we analyzed a 770 gene “pan-cancer” panel together with 31 AOM-DSS-induced (23 FFPE and 8 frozen) and 25 *Apc*^*fl*^;Lrig1-CreER^T2^ (12 FFPE and 13 frozen) Oct1 wild-type samples. The total output data is shown in S1 Table. Supervised clustering and applying the same FDR and fold-change cutoffs identified a group of 111 differentially expressed genes. These are shown in S1 Table.

**Fig 8:**
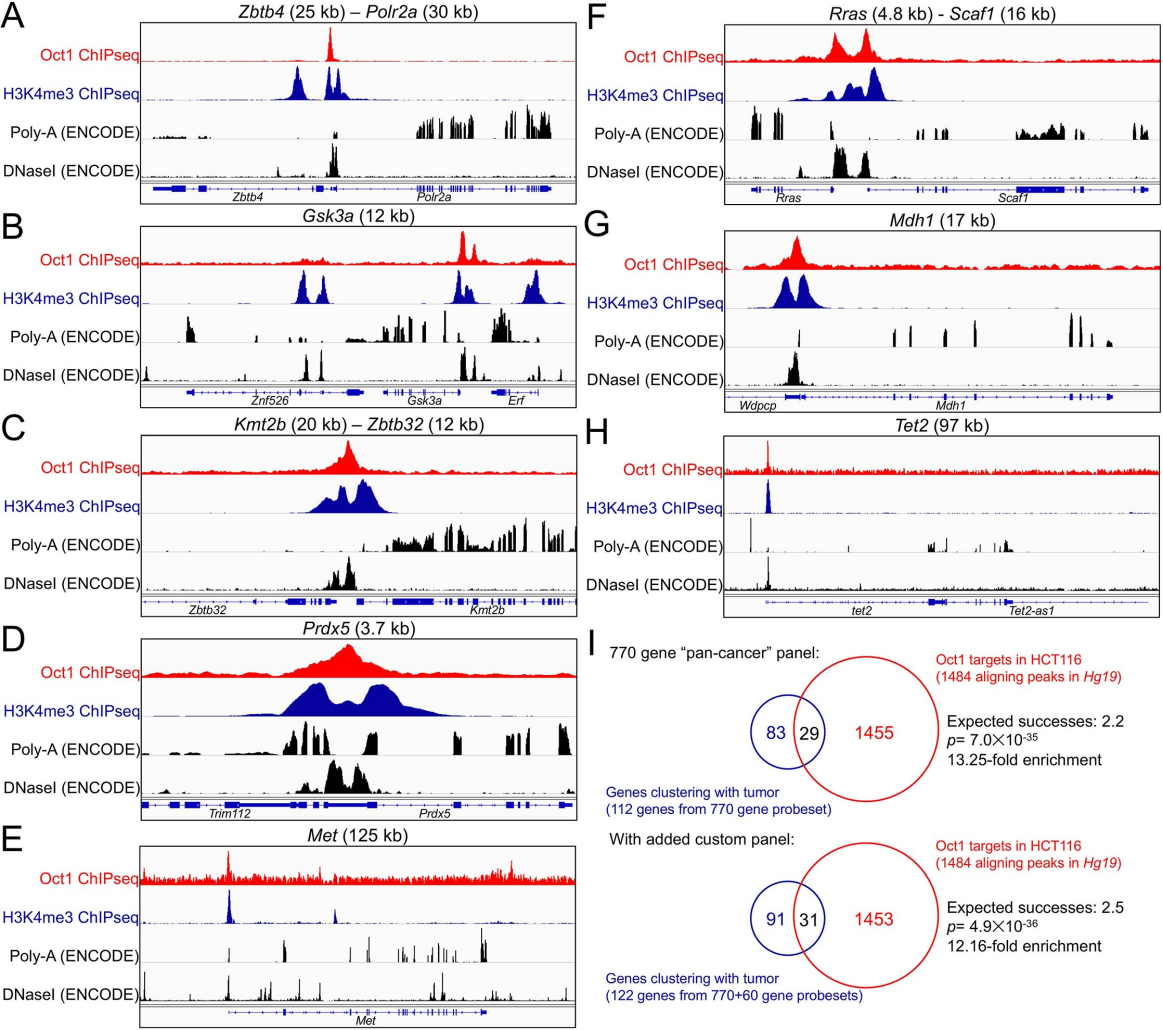
Putative Oct1 targets in human HCT116 cells significantly overlap with mouse genes differentially expressed between the two tumor models. (A) Example tracks (*Hg19*) are shown of Oct1 (red) and H3K4me3 (blue) ChIPseq using human HCT116 cells. The region of chromosome 17 corresponding to the *Zbtb4* –*Polr2a* locus is shown. In black is shown ENCODE HCT116 RNAseq and DNaseI hypersensitivity at the same regions. (B) Similar data for *Gsk3a*. (C) *Kmt2b* –*Zbtb32*. (D) *Prdx5*. (E) *Met*. (F) *Rras*–*Scaf1*. (G) *Mdh1*. (H) Tet2. (I) Intersection of genes enriched from a PanCancer Pathways probeset with Oct1 targets identified by ChIPseq in HCT116 cells. A second analysis is also shown that includes additional genes enriched in a smaller custom probeset (S5 Table).

We previously identified Oct1 target genes in T cells and embryonic stem cells [6, 51]. To identify targets in the context of colon cancer, we used Oct1 ChIPseq together with human HCT116 cells. HCT116 cells have the advantage of extensive profiling by the ENCODE (ENCyclopedia Of DNA Elements) consortium, including for attributes such as gene expression and DNaseI hypersensitivity [52]. We performed Oct1 and H3K4me3 ChIPseq using HCT116 cells, and input controls, identifying 1484 high-quality Oct1 peaks (see methods). Comparing these peaks to DNaseI-hypersensitivity data from the ENCODE consortium showed greater than 26% (398) of the 1484 Oct1 peaks intersect with DNaseI-hypersensitive peaks with no gap (*p*<0.001, S2 Table). Profiling likely target genes near these peaks (<20 kb) identified 847 genes (S3 Table). Examples are shown in Fig 8. The gene associated with the strongest peak in this analysis (S3 Table) is *Zbtb4* (Fig 8A). The product of this gene is associated with the mitotic checkpoint [53]. *Zbtb4* deletion in mice results in aneuploidy [53]. Other shown putative target genes are *Gsk3a*, *Kmt2b*, *Prdx5*, *Met*, *Rras*, *Mdh1* and *Tet2* (Fig 8B–8H). Analyzing pathways enriched in the gene list identified 127 terms with *p*<0.05 (S4 Table). The top five pathways in this analysis were all heavily influenced by the presence of the multiple histone clusters as Oct1 targets (S3 Table), e.g., “nucleosome” was the top enriched pathway with 19 genes. Other identified pathways include “cell division” (#11, 10 genes), “spindle” (#17, 6 genes), “mitosis” (#18, 7 genes), “regulation of stem cell differentiation” (#26, 2 genes), “beta catenin binding” (#31, 3 genes) and “regulation of Notch signaling pathway” (#60, 2 genes). These pathways are consistent with roles for Oct1 in transcription stem cells, malignancy and mitotic regulation.

We compared the Oct1 targets identified in human HCT116 cells with the set mouse genes whose expression clusters with tumor type. Of the ten genes identified in the custom probeset whose expression correlates with the mouse tumor model (FDR<0.05, fold change>1.3), two (*Lin28a* and *Lrig1*) are within 20 kb of an Oct1 ChIPseq peak in HCT116 cells (S5 Table). Similarly, of the 112 identified genes in the “pan-cancer” probeset whose expression correlates with the mouse tumor model, 29 correspond directly to human genes within 20 kb of an Oct1 peak (e.g., *Stat3*, *Bax*, *Tgfbr2*, *Tet2*, *Vegfa*, *Jag1*, S5 Table). The number of differentially expressed genes that are in the ChIPseq dataset is greater than what would be expected by chance (Fig 8I). Combining the two datasets (31 genes) also shows significant over-representation in the intersection between differentially expressed genes and putative Oct1 targets (Fig 8I). Collectively, these results indicate that genes that tend to be differentially expressed between tumors from the two models are enriched for nearby Oct1 target sites.

## Discussion

Here we show that the transcription factor Oct1 is dispensable for mouse gut epithelial cell homeostasis, but is essential for recovery of the colon following damage in vivo, and for passage of intestinal organoids in vitro. Colonic epithelium lacking Oct1 maintains normal architecture for up to 150 days, consistent with an interpretation that Oct1 is dispensable for maintenance of the normal gut. In contrast to homeostatic conditions, Oct1 is required to recover the colon epithelium following DSS exposure, a situation that requires recognition of tissue damage followed by a high level of proliferation. Either a defect in proliferation and regeneration of the colon epithelium, an increase in cell death during this process, or both could underpin these findings. The findings are consistent with prior work from others indicating that homeostasis and regeneration are molecularly and physiologically distinct [54–58], and with work showing that Oct1 becomes phosphorylated following stress exposure and associates with target genes containing a variant DNA binding site known as a MORE [5]. Example genes include *Ahcy*, *Blcap*, *Zmiz2*, *Rras*, *Rras2*, *Bmp4* and *Abcb1* [5, 9]. Other work in DLD-1 colon cancer cells shows that Oct1 rapidly changes transcriptional cofactors in response to MAPK signals at the target gene *Cdx2* [7].

Intestinal organoids have been used before to study regeneration [59]. Consistent with the finding that Oct1 loss did not affect small intestinal homeostasis in vivo, morphologically normal organoids could be directly explanted from tamoxifen treated mice. Interestingly, unlike control organoids we found that intact organoids lacking Oct1 could not be maintained by passage in vitro. Instead, the isolated crypts structures could close but did not generate new villus and crypt domains. The underlying causes may be similar to those underlying the phenotype of DSS-treated mice in vivo, though more study is required to test this idea.

**Fig 9:**
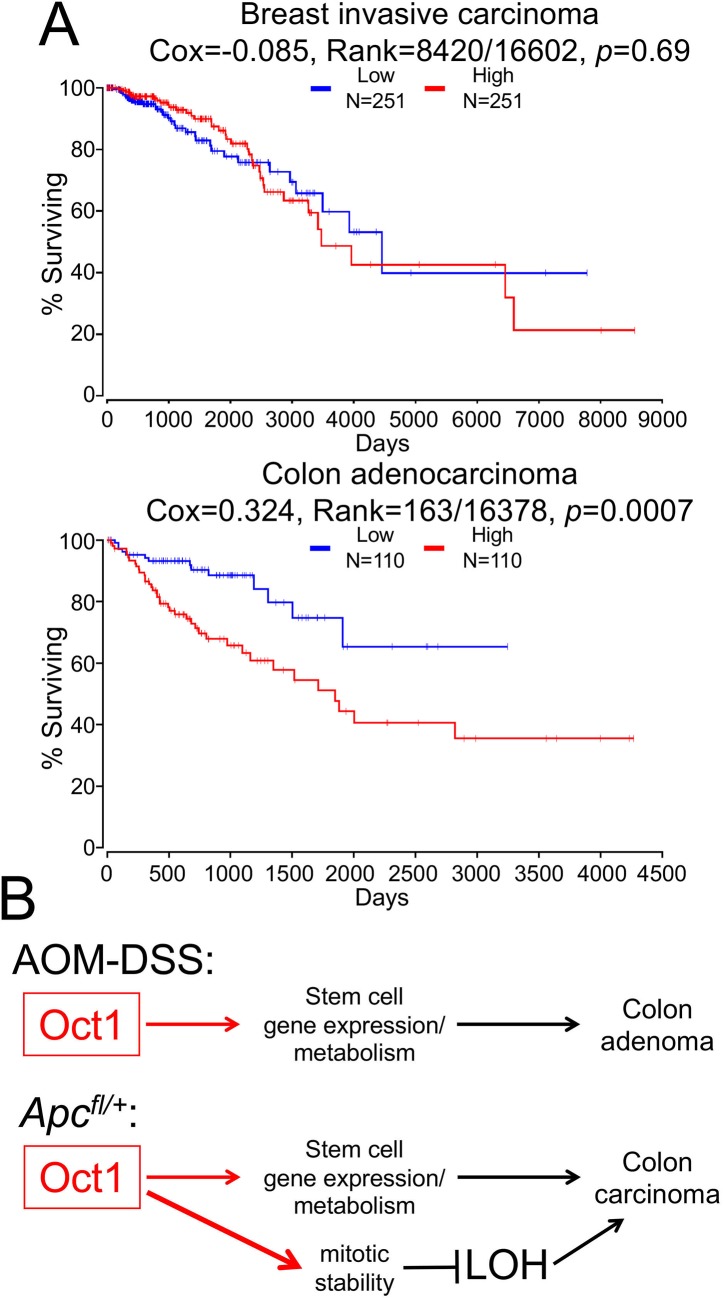
Effect of *Oct1* mRNA expression level in human colon and breast cancer, and model for Oct1 function in different mouse models of gut malignancy. (A) Kaplan-Meier survival curves were downloaded and modified using OncoLnc [69] and gene symbol *Pou2f1*. Survival in days of human breast and colon cancer patients in whose tumors *Oct1* (*Pou2f1*) mRNA expression falls in the bottom (“Low”) or top (“High”) quartiles of the group as a whole is shown. (B) Model of Oct1 functions in the AOM/DSS vs. *Apc*^*fl/+*^;Lrig1-CreER^T2^ colon tumor models. In this model, Oct1 in tumors based on *Apc*^*fl/+*^ LOH gains a more dominant tumor suppressive activity by promoting mitotic stability.

We also show that Oct1 loss has potent effects on tumorigenicity in two different mouse models of colon malignancy. Using an AOM-DSS model, Oct1 loss strongly protects mice from tumors. Elevated Oct1 mRNA expression is a negative prognostic factor in colon but not breast cancer (Fig 9A). The normal appearance of the colon following Oct1 deletion, coupled with the protection afforded by Oct1 loss, suggests a “therapeutic window” in which targeting Oct1-associated pathways could be used to treat certain GI malignancies with minimal side effects. More study will be required to determine the conditions in which targeting Oct1 and its associated pathways in gut malignancy could be beneficial. In a second model driven by *Apc* LOH (*Apc*^*fl/+*^;Lrig1-Cre), Oct1’s dominant activity is tumor suppressive, with more tumors of equal grade generated. This finding, together with the equivalent amount of pH3 staining in the tumor sections, suggests an increase in tumor initiation though not of progression. The molecular mechanisms underlying the differences in the two tumor models may involve changes in gene expression and/or a role for Oct1 in mitosis (below). Of note, similar opposing roles were recently described for the *Drosophila* Oct1 ortholog, Nubbin, based on its different isoforms [60]. Different mammalian Oct1 isoforms have been described [61, 62], and thus Oct1 may regulate normal and malignant epithelial tissue states through evolutionarily conserved mechanisms.

In HeLa cells and MEFs, Oct1 loss slightly increases the rate of mitotic chromosome segregation abnormalities, resulting in increased lagging chromosomes and aneuploidy [4]. Consistent with these prior findings, Oct1 loss accelerated tumorigenesis and increased tumor number in a colon cancer model driven by LOH of the tumor suppressor gene *Apc* in Lrig1^+^ cells [35]. In addition to increased LOH, differences in the molecular pathways and vulnerabilities associated with Oct1 in the two tumor models could contribute to the difference. To test this idea, we profiled gene expression in AOM-DSS tumors and tumors in which *Apc* is deleted in Lrig1^+^ cells, identifying a set of differentially expressed genes that include *Lef1*, *Ldha*, *Lrig1*, *Tet2*, *Atm* and *Bax*.

We performed ChIPseq using HCT116 colon carcinoma cells, identifying ~850 putative target genes associated with metabolism (e.g., *Prdx5*, *Mdh1*, *Ahcy*), transcription (*Taf12*, *Tet2*, histones), malignancy (*Met*, *Blcap*, *Rras*, *Jag1*, *Gsk3a*) and mitotic stability (*Zbtb4*). The set of targets includes some genes (*Ahcy*, histones, *Blcap*, *Rras*) identified previously in other tissues [5, 51], as well as others (*Prdx5*, *Tet2*, *Mdh1*, *Jag1*, *Gsk3a*) unique to this analysis. Intersection of the Oct1 target gene set with the set of genes differentially expressed in the two tumors types reveals a significant enrichment. Examples include *Lin28a*, *Lrig1*, *Tet2*, *Vegfa* and *Jag1*. Either mitotic regulation or differential regulation of gene expression by Oct1 (or both) could therefore explain the opposing effects of Oct1 loss in the two models. A model for the functions of Oct1 in the two tumor models is shown in Fig 9B. In this model, Oct1 promotes AOM/DSS-induced tumors through actions on target genes controlling metabolism and stem cell identity. Because Oct1 promotes mitotic stability and because Oct1 target genes are differentially expressed in the more aggressive *Apc*-LOH model, Oct1 instead acts as a tumor suppressor. Cumulatively, the findings indicate that Oct1 is a potent regulator of colon malignancy, but that its functions are dictated by the colon tumor model used.

## Materials and methods

### Ethics statement

All *in vivo* experiments were reviewed, approved, and conducted in compliance with the University of Utah’s Institutional Animal Care and Use Committee and the NIH Guide for Care and Use of Laboratory Animals guidelines. Animal protocol: 17–05008. Animal anesthesia and euthanasia involved the application of isofluorane.

### Laboratory mice

All mice used in this study were mixed C57BL/6:129/Sv background. The Oct1 conditional allele has been described previously [6]. The Lrig1-CreER^T2^ mouse allele [35] was a gift of Robert Coffey (Vanderbilt). The Lgr5-EGFP-IRES-CreER^T2^ allele [36] was purchased from Jackson labs. *Apc*^*loxp exon14*^ *(Apc*^*fl*^*)* has been previously described [48] and was a gift from Ömer Yilmaz (MIT). Food and water were available *ad libitum*. Tamoxifen (200 μL 10 mg/mL in corn oil) was administered by oral gavage. All mice were treated at 6–8 weeks of age. Regeneration and azoxymethane (AOM) chemical tumorigenesis experiments used a single tamoxifen treatment. The *Apc* tumor model received three treatments on consecutive days. Dextran sodium sulfate (DSS, MP Biomedicals) was provided in drinking water at a concentration of 2.0% for AOM-induced tumors, and 2.5% for regeneration. AOM (Sigma) was provided by IP injection (10mg/kg). AOM-DSS treatments followed the protocol in [43].

### Immunoblotting

Antibodies used for immunoblots were as follows: Oct1, Bethyl #A310-610 (1:1000); β-actin, Santa Cruz #sc-47778 (1:1000).

### Preparation of colon crypts for immunoblotting

Colon crypts were isolated as previously described [36, 50, 63], with modifications. Briefly, small colon fragments were incubated in PBS with 4mM EDTA for 30 minutes at 4°C, followed by vigorous shaking. Colon crypts were collected after passing through a 70 μm cell strainer and incubated in lysis buffer for 30 minutes on a rotator at 4°C. After centrifugation at 400 × *g* for 10 minutes to remove cell debris, the lysates were prepared by boiling in 4X sample buffer.

### Histological scoring and immunohistochemistry

H&E stained sections were reviewed by a gastrointestinal pathologist and assessed for features of dysplasia including cytologic atypia, architectural complexity, and invasive tumors. Scoring criteria were as follows, with no tumors scoring higher than grade 2: grade 1: dysplastic epithelium with simple glandular architecture, with intervening lamina propria between the glands; grade 2: dysplastic epithelium with complex, crowded glandular architecture with cribriform glands, and lack of intervening lamina propria. IHC was performed as in [21]. The slides were developed with DAB peroxidase substrate (Vector Laboratories, SK-4100) as per manufacturer instructions, and were counterstained with hematoxylin. After dehydration (3 min washes each of 70%, 85%, 95% and 100% ethanol) the slides were incubated in xylene for 3 min twice and mounted using Limonene mounting medium (Abcam #104141). Antibodies used for IHC were as follows: Oct1, Abcam #178869 (1:250), total β-catenin, Santa Cruz #8814 (1:300), phospho-histone H3, Abcam #5176 (1:200).

### Immunofluorescence

Paraffin-embedded tissue slides were rehydrated by incubation in xylene for 4 minutes, followed by xylene, 100%, 95%, 85% and 70% ethanol for 3 minutes each. Antigen retrieval was performed in 10mM sodium citrate buffer, pH 6.0 in a steamer. Slides were blocked in PBS + 3% BSA for 1h at RT, followed by primary antibody incubation at 4°C overnight. Antibodies used for IF staining were as follows: Lrig1, Thermo Scientific #PA547009 (10 μg/ml) and GFP, Abcam #290 (1:500). Images were collected and analyzed using an Axio Observer Z1 imaging system (Carl Zeiss).

### Microbial infiltrate

To determine gut barrier defects following DSS-induced damage of the colon, spleens were harvested at end-point and placed into 15 mL conical tubes containing 5 mL of PBS on ice. Spleens were homogenized for 5 seconds using a tissue homogenizer (Tekmar). 10 μL of undiluted homogenate was plated on 5% sheep blood agar plates (Thermo Scientific #R01200), in parallel with 10 μL of serial 1:10 dilutions to generate a colony counts over multi-log range. Plates were incubated at 37°C overnight. CFUs were counted and the total number of microbes calculated using the dilution factor.

### Intestinal organoids

Organoids were maintained as previously described [50, 63] with modifications. Crypts from tamoxifen-treated or untreated mice were plated in 8-well chambered slides in 40μl of Matrigel at a density of ~40 crypts per Matrigel droplet. Organoids were grown for 5–7 days until fully grown. Mature organoids were passaged every 5–7 days. Images were taken using an Olympus IX-50 inverted microscope. Quantifications were performed using Image J software (National Institutes of Health).

### Tumor gene expression profiling

Gene expression was measured in Oct1 wild-type formalin-fixed, paraffin-embedded (FFPE) and snap-frozen tumors using NanoString (Seattle, WA). For frozen samples, tumors were dissected from the normal mucosa under an Olympus SZ61 dissecting microscope in ice cold PBS and snap frozen in liquid nitrogen. Two gene panels were used: the 770-gene “pan-cancer” panel (23 FFPE AOM-DSS tumors and 12 FFPE *Apc*^*fl/+*^;Lrig1-CreER^T2^ tumors), and a custom 60 gene panel enriched for gut stem cell-associated genes [50] (23 FFPE and 8 frozen AOM-DSS tumors, and 12 FFPE and 13 frozen *Apc*^*fl/+*^;Lrig1-CreER^T2^ tumors). Laser capture microdissection was performed by the Molecular Pathology Core at the University of Utah. Total mRNA was purified with using a Qiagen miRNeasy FFPE Kit (217504). Analysis and normalization of the raw data were conducted with nSolver Analysis Software v4.0 (NanoString Technologies). Genes whose expression significantly correlated with tumor type were identified using Prism GraphPad Row Statistics. Multiple T-tests were performed on each gene individually, without assuming a consistent standard deviation. Cutoffs were determined using the Two-stage linear step-up procedure of Benjamini, Krieger and Yekutieli [64], with *Q* = 5%, and a linear fold change less than 0.769 or greater than 1.3 fold. For each differentially expressed gene set, ascription of statistical significance for Oct1/Oct4 target gene enrichment was made using a hypergeometric test.

### ChIPseq

Human HCT116 cells (ATCC) were cultured in DMEM supplemented with 5% calf serum (Sigma), 5% fetal calf serum (X&Y Cell Culture), 100 U/ml penicillin, 100 μg/mL streptomycin and 2 mM L-glutamine (Invitrogen). Cells were screened monthly for mycoplasma by PCR. Cells were cultured at 37°C in a humidified atmosphere containing 5% CO_2_. Cells were prepared for ChIPseq as previously described [65] with modifications. Briefly, protein-DNA complexes were cross-linked with 1% formaldehyde for 8 minutes, followed by quenching with 0.125M glycine for 5 minutes. Sonication was performed using the EpiShear Cooled Sonication Platform (ActiveMotif #53080) with 10 pulses consisting of a 20-second sonication followed by a 30-second rest at 25% amplitude on tube coolers to yield a range of products between 200–500 bp. Sonicated chromatin was collected after centrifugation at 16,000 × *g* for 5 min at 4°C. Immunoprecipitation used Oct1 (Bethyl #A310-610A) or H3K4me3 (Cell Signaling #9727) antibodies. Cross-links were reversed overnight at 65°C, followed by purification of the enriched DNA using a Qiagen Qiaquick PCR cleanup kit (Cat # 28104). For both Oct1 and H3K4me3 ChIPseq, three biological replicates of sub-confluent HCT116 cells were performed, with three input controls. Library preparation was performed using an NEBNext ChIP-Seq Library Prep Master Mix Set with Unique Dual Index Primers. Paired-end sequencing took place on an Illumina NovaSeq platform. For sequence analysis, paired end fastq datasets for each sample were aligned to the B37 human reference using bowtie2 [66], sorted, and duplicates removed with Picard tools (https://broadinstitute.github.io/picard/). Uniquely mapped, duplicate free, paired end alignments were merged and their center positions extracted using USeq [67]. Both replica specific and merged replica ChIPSeq peak identification was performed following the USeq ChIPseq protocol (http://bioserver.hci.utah.edu/USeq/Documentation/usage.html) with methodology detailed in [67]. ChIPseq peaks of moderate and high stringency were identified by setting both an FDR and log2 Ratio threshold; FDR of > = 0.05 and log2 ratio > = 0.585 for moderate, FDR of > = 0.01 and log2 ratio > = 1 for high. Peaks intersecting satellite repeat regions were excluded. This analysis resulted in between 55 and 105 million paired-end sequence reads (39 and 55 million merged reads), 77 to 86% of which uniquely aligned to the human B37 reference genome. Merging the datasets from the three replicates identified 1484 high-quality Oct1 peaks (FDR>.01, log2 fold enrichment>1). Pathway analysis was performed using the BaseSpace Correlation Engine (Illumina).

### Data availability

Raw and processed data have been made available in the Gene Expression Omnibus (GEO) public repository (GEO series record GSE123513).

### Statistical analyses

Student’s T-tests were used throughout to determine *p*-values. Error bars denote ±standard deviation except where noted. A single * indicates *p*<0.05, ** indicates *p*<0.01, *** indicates *p*<0.001. ChIPseq *p*- and *q*-values were determined using a binomial distribution and multiple test correction controlled using Storey *q*-value/FDR [68]. For survival analysis, *p*-values were calculated using a logrank test.

## Supporting information

S1 Table Genes with expression correlating to tumor model (AOM-DSS vs Apc-LOH;Lrig1-CreER). (XLSX)

S2 Table Overlap between HCT116 Oct1 ChIPseq peaks and prior HCT116 DNaseI hypersensitive sites (ENCODE). (XLSX)

S3 Table Nearest genes to Oct1 ChIPseq peaks using Fdr cutoff 20/distance cutoff of 20kb. (XLSX)

S4 Table Pathway analysis of genes nearby Oct1 target sites. (XLSX)

S5 Table Intersection between Oct1 human ChIPseq and mouse genes differentially expressed in the chemical vs LOH models. (XLSX)
